# Supernumerary Phantom Limb After Stroke in the Left Hemisphere: A Case Report

**DOI:** 10.7759/cureus.66681

**Published:** 2024-08-12

**Authors:** Takumi Matsuyama, Koji Hayashi, Yuka Nakaya, Asuka Suzuki, Yasutaka Kobayashi, Mamiko Sato

**Affiliations:** 1 Department of Rehabilitation Medicine, Fukui General Hospital, Fukui, JPN; 2 Graduate School of Health Science, Fukui Health Science University, Fukui, JPN

**Keywords:** unilateral spatial neglect (usn), stroke, phantom limb, phantom limb sensation, supernumerary phantom limb

## Abstract

Supernumerary phantom limb (SPL) is a rare sensation of additional limbs that are perceived to exist alongside existing limbs. SPL can occur in various neural regions, but it is rare for SPL to be caused by left hemisphere cerebral infarction. In this report, we describe a case of a 64-year-old woman with SPL after a stroke. She had a history of handedness change. A neurological examination revealed that she had right hemiplegia, unilateral spatial neglect (USN), anosognosia, and pan-sensory loss on the right side of her body. Brain magnetic resonance (MR) imaging disclosed cerebral infarction in the left corona radiata region. She suffered from SPL in the right upper limb. Although SPL was prolonged, the recovery of USN was noted four months after onset, followed by the improvement of SPL.

## Introduction

A phantom limb is an illusion where an amputated limb feels as if it is still present, a phenomenon experienced by more than 90% of amputees [1]. However, in rare cases, people without limb defects can experience sensations of additional limbs that are not present alongside their existing limbs. This phenomenon has been named supernumerary phantom limb (SPL) or phantom third hand by Critchley [2]. The reports about SPL are very limited, and Kim et al. summarized only 28 cases in their study [3]. The location of lesions associated with SPL is diverse and has been reported in the frontal lobe, temporoparietal lobe, temporal lobe, basal ganglia, cerebellum, pons, upper cervical spinal cord, and peripheral nerves [3]. In this report, we describe a case of SPL with right hemiparalysis, loss of superficial and deep sensation, unilateral spatial neglect (USN), and anosognosia after left cerebral infarction.

## Case presentation

**Figure 1:**
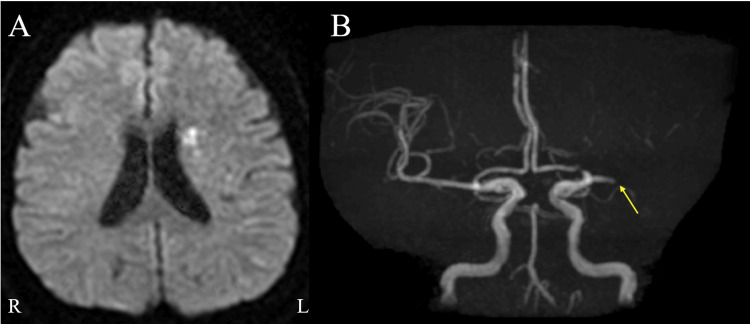
The results of brain MR imaging on admission (A) Diffusion-weighted brain MR imaging showing hyperintensity in the left corona radiata region. (B) MR angiography showing occlusion of the left middle cerebral artery (arrowhead).

**Figure 2:**
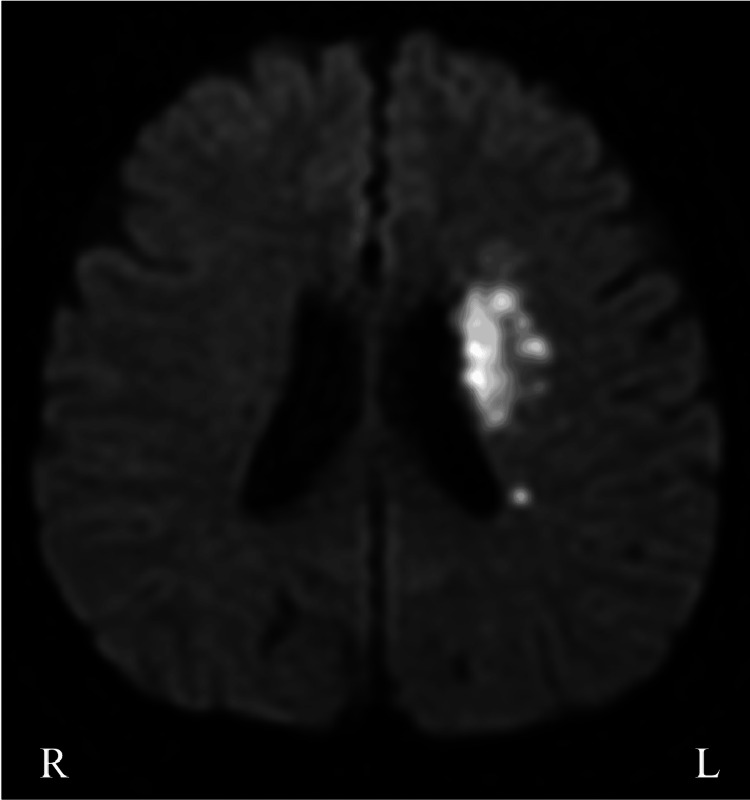
The result of brain MR imaging three days after admission The third-day brain MR imaging showing an increase in DWI infarct volume.

A 64-year-old woman suddenly developed right hemiplegia and was transported to our hospital. She had a history of smoking 20 cigarettes a day, angina pectoris, hypertension, and hyperlipidemia, but she had discontinued visiting the hospital. She was naturally left-handed but had been corrected to become right-handed. On admission, vital signs were unremarkable except for high blood pressure (240/130 mmHg). Neurological examination showed no facial paralysis but revealed the Medical Research Council (MRC) grade 4 muscle weakness in the right limbs. No sensory disturbances were pointed out on admission. Diffusion-weighted brain magnetic resonance (MR) imaging disclosed hyperintensity in the left corona radiata region, and MR angiography revealed an occlusion of the left middle cerebral artery (Figures 1A, 1B). We diagnosed atherothrombotic cerebral infarction and treated her with argatroban (60 mg/day for the first two days, followed by 20 mg/day for the next five days) and edaravone, but on the third day, her neurological symptoms worsened, manifesting as USN, anosognosia, MRC grade 1 or 2 muscle weakness in the right limbs, and pan-sensory loss in both superficial and deep sensation. Aphasia did not develop. Additionally, she suffered from SPL on the affected side. She reported a loss of sense of belonging to the affected limb and made comments suggesting delusional misidentification. Specifically, the patient reported experiencing a phenomenon in which she perceived multiple paralyzed arms. She described sensations akin to her arms melting away, feeling many right hands on her face, and having several children’s arms entangled around her arm. In her perception, these arms seemed to occupy different spatial locations and were not her own. SPL was exacerbated on the bed at night. Additionally, she developed right arm and back pains. A restudy of brain MR imaging revealed extended areas of hyperintensity on diffusion-weighted imaging compared to the findings on admission (Figure 2). Although cognitive assessments, including the Revised Hasegawa Dementia Scale (HDS-R: 29 points) and Mini-Mental State Examination (MMSE: 24 points), were preserved, the frontal assessment battery (11 points) and the Japanese version of the Trail Making Test (part A: 229 sec; part B: >300 sec) were affected. In tests such as the letter cancellation task and star cancellation task, the patient consistently missed all items on the right side. We planned rehabilitation treatments, including conventional physical and occupational therapies for paralysis, alongside visual compensation for USN and psychiatric treatments for hallucinations and delusions.

Our patient experienced USN, anosognosia, severe right hemiplegia, and sensory impairment in the right upper and lower limbs, which persisted. However, approximately three months after onset, there was an increase in time spent out of bed, enabling an active implementation of a top-down approach to the right USN. This led to heightened awareness toward the paralyzed limb and mild improvement in the right USN. Around four months after onset, complaints of pain began to diminish, and there was a decreasing trend in the frequency of reports of SPL. By approximately five months after onset, complaints of pain or SPL had nearly disappeared.

## Discussion

This report describes a case of SPL caused by a stroke. The patient presented with USN, anosognosia, and pan-sensory loss affecting both superficial and deep sensation, in addition to right hemiplegia. In relation to SPL, she experienced sensations of having multiple arms and a loss of ownership of these arms. Interestingly, SPL gradually disappeared depending on the recovery of USN.

While reported cases of SPL in cerebrovascular disorders are rare [4], one study noted that SPL after stroke is relatively common but often underreported; 27 of 50 stroke patients developed SPL [5]. In the latter report, SPL is divided into two types: postural phantoms and kinesthetic phantoms. The former involves patients being perceived as illusions of limb position, which often occur while lying in bed at night, a period when visual input is absent from multi-sensory integration. The latter develop illusory movements ranging from simple single-joint sensations to complex goal-directed phantom movements. In addition, SPL primarily occurs in right hemisphere disorders, but it can also arise in left hemisphere disorders [5,6]. This may be influenced by each patient’s natural handedness. In addition, several parts of the brain can be related to SPL. Kim et al. described a case of right basal ganglia hemorrhage associated with SPL [3]. This case had disturbances in the superficial and deep sensation and left-side homonymous hemianopia. Yoo et al. described two cases of SPL associated with pontine hemorrhage [7]. All two cases had superficial and deep sensory disturbances and right-side USN or hemianopia. The authors mentioned one case with exacerbation of SPL during the night while lying in bed [7]. Hari et al. described a case of SPL after an aneurysm rupture with neonatal partial callosal disconnection and postoperative infarction of the medial frontal cortex [8]. Neurological examinations in this patient showed no sensory or visual disturbances but revealed tactile anomia in the left hand and left-hand dominant constructional apraxia. She also developed both SPL and alien hand syndrome. In other body parts, cervical spinal cord injuries [2,9] and peripheral neuropathies [10] have been reported in relation to SPL. Critchley, who first used the term SPL and phantom third hand, described SPL as occurring after injury to the anterior roots of C3 to C7 and the posterior roots of C3 to C6, as well as involving the brain [2]. Although little detailed examination of the brain has been conducted in this case, the authors emphasize that in addition to damage to the peripheral nervous system, brain damage is also significant in causing the onset of SPL. However, there are cases where brain lesions are not clear in SPL following cervical spinal cord injury [9]. Melinyshyn et al. reported three cases of acute inflammatory demyelinating polyneuropathy with SPL [10]. All three cases had sensory disturbance, and one case noted exacerbation at night [10].

The mechanisms behind the development of SPL remain unknown, but a few pathways can be suggested. It is reported that deafferentation of the sensory system is an important requirement for the manifestation of phantom limbs [3]. Damage to subcortical structures impairs sensory functions, such as proprioception, kinesthesia, and touch. Despite this, efference copies of motor commands continue to be output from preserved cortical areas. Meanwhile, sensory feedback from the periphery diminishes. As a result, a dissociation occurs between motor output and sensory input, leading to a distorted bodily sensation known as phantom limb sensation [3]. In addition, it is noteworthy that many reports, including our case, have observed the coexistence of USN and/or anosognosia [3,7,11]. In our case, SPL was diminished depending on the improvement of USN. Moreover, especially in cases of postural phantoms, there is no doubt that visual information is one of the triggers of SPL when considering the onset of SPL at night with the absence of visual information.

Additionally, previous functional MRI studies have been conducted in cases of SPL. Khateb et al. evaluated SPL that not only could be moved but also perceived visually and felt upon touch, using fMRI, in patients with lesions in the right insula [12]. Their results showed activation of multiple brain regions, including the motor cortex, premotor cortex, visual cortex, and somatosensory cortex, supporting the patient’s complaints. They discussed that this cortical hyperactivity resulted from centripetal pathway disruption due to subcortical damage [12]. In another report, McGonigle et al. revealed high activity on the right medial wall in the supplementary motor area [13] in a case previously described by Hari et al. [8]. As this case had not only SPL, especially kinetic phantom, but alien hand syndrome, and the supplementary motor area is associated with alien hand syndrome [14], we assume that it is difficult to generalize for all SPL cases.

In our case, the pattern of SPL was classified into postural phantom. She had USN and anosognosia in addition to pan-sensory loss. We speculated that the involvement of both sensory feedback from the periphery and visual feedback may develop SPL. In addition, our patient developed SPL after a left cerebral infarction. It has been reported that SPL is often associated with damage to the right cerebral hemisphere. However, in this case, due to the patient’s history of handedness correction, it is likely that the dominant hemisphere of the cerebral cortex had been reversed. Indeed, despite the stroke involving the left cerebral language areas, the patient did not develop aphasia. When cerebral dominance is reversed, left hemisphere damage can result in SPL.

## Conclusions

We presented a case of SPL after left cerebral infarction. The patient may develop SPL due to visual disturbances, including USN and anosognosia, as well as pan-sensory loss. SPL due to damage to the left cerebral hemisphere is rare but may result from handedness switching. Our report covers a single case, and it is necessary to accumulate more cases to reveal the underlying mechanism of SPL development.
